# A Novel Polymeric Adsorbent Embedded with Phase Change Materials (PCMs) Microcapsules: Synthesis and Application

**DOI:** 10.3390/nano9050736

**Published:** 2019-05-13

**Authors:** Xingang Li, Lingyu Sun, Hong Sui, Lin He, Wei Yuan, Zhenwei Han

**Affiliations:** 1 School of Chemical Engineering and Technology, Tianjin University, 92 Weijin Road, Tianjin 300072, China; lxg@tju.edu.cn (X.L.); sunnixo@163.com (L.S.); suihong@tju.edu.cn (H.S.); hanzhenwei@tju.edu.cn (Z.H.); 2 National Engineering Research Center of Distillation Technology, 92 Weijin Road, Tianjin 300072, China; 3 Collaborative Innovation Center of Chemical Science and Engineering, 92 Weijin Road, Tianjin 300072, China; 4 College of Chemistry, Liaoning University, 66 Chongshan Middle Road, Shenyang 110036, Liaoning, China

**Keywords:** polymer, phase change materials, microcapsule, adsorption, heat storage

## Abstract

The heat released during the industrial gas adsorption (e.g., volatile organic compounds (VOCs)) on adsorbents (e.g., activated carbon) would lead to the risks of fire and explosion in the adsorption column. Herein, a novel highly-porous Vinylbenzyl chloride-Divinylbenzene (VBC-DVB) polymeric adsorbent was synthesized with embedded microcapsules (Hypercrosslinked VBC-DVB Beads (HVPM)). These microcapsules have a polydivinylbenzene-phase change materials (DVB-PCMs) core-shell structure. Paraffin wax was used as PCM filling in the spherical capsule. This microcapsules-embedded polymeric adsorbent HVPM (Φ1.5–2.0 mm) is found to possess a high specific surface area (~665 m²/g) and micropore-dominant structure. It also has heat storage capability indicated by DSC (Differential Scanning Calorimetry) analysis (11.1 J/g heat of fusion between 35.0 and 48.2 °C) for the encapsulated paraffin wax. The lab adsorption tests proved the capabilities of HVPM in adsorbing VOCs (toluene, 0.21 g/g) and controlling the temperature inside the adsorption column during the dynamic adsorption process, in which the temperature rise was lowered by 62.5%, relatively.

## 1. Introduction

Volatile organic compounds (VOCs) are one of the most important air pollutants, because of several harmful effects on human health and environment [1]. Different technologies are available to reduce VOCs emissions including adsorption, condensation, thermal oxidation, catalytic oxidation, biofiltration, and photocatalytic oxidation [2,3,4,5]. Among these technologies, adsorption is considered as the promising way to treat the industrial waste organic gases, due to its wide range of applicability and high effectiveness. The activated carbon, with high specific surface area and hydrophobicity, is the most used adsorbent for VOCs in the adsorption industry. However, during the gas adsorption by activated carbon (especially for the high concentration VOCs), a large amount of heat is often released, leading to the increase of the temperature in the adsorption column. The increased temperature in the column could reach 50 °C, which would cause the risk of fire or explosion [6]. In addition, the adsorption efficiency would be lowered because of the increased temperature [7]. Therefore, controlling the temperature during the adsorption is an important consideration in future industrial applications. 

The hypercrosslinked polymeric adsorbents (HPAs), organic materials with micropore-dominant structure, are prepared by the extensive crosslinking of linear or lightly crosslinked precursor polymers [8]. They have high Brunner−Emmet−Teller (BET) surface area, comparable adsorption capacities with activated carbon for VOCs, and stable physical and chemical properties [9]. The HPAs are produced by an intermolecular and intramolecular crosslinking reaction with the Friedel–Crafts alkylation reaction, radical polymerization, or organolithiation routes [10,11,12]. These HPAs have been widely applied in air, water purification processes, and gas adsorption [13]. Herein, it is our attempt to apply the HPAs or derivatives as the adsorbent materials together with microencapsulated phase change materials. 

Phase change materials (PCMs) can store and release a large amount of energy through their phase change, keeping the temperature in a stable state [14]. PCMs have wide applications in thermal energy storage [15] and environment protection [16]. Paraffin waxes are the most commonly used PCM because of low cost, high energy storage density, and largescale availability [17,18]. Microencapsulation refers to the formation of small, coated particles loaded with material in a certain phase, such as solid, liquid, solid/liquid dispersion, or gas [19]. Microcapsules loaded with phase change materials (MPCMs) can store a large amount of thermal energy and can be used circularly. Since the encapsulated PCMs are protected from air, moisture, microorganisms, and other contaminants, spoilage is reduced and the shelf-life of PCMs is increased. Nowadays, MPCMs have been successfully used in a wide range of applications. Cabeza et al. used MPCMs in concrete walls for energy savings [20]. Younsook et al. treated polyester knit fabrics with MPCMs to develop thermoregulating textile materials [21]. Hoang et al. validated the thermal buffering capacity of MPCMs in food packaging application [22]. These examples provide us with an idea of the high potential MPCMs in the adsorption industry to solve the issue of rising temperatures. In this study, we aim to embed microcapsules containing paraffin wax in a hypercrosslinked polymeric adsorbent to help control temperature in the adsorption column. 

Microcapsules and polymeric adsorbent share a similar route of synthesis called “suspension polymerization” [23,24]. A typical suspension polymerization system includes an oil phase, consisting of a monomer, an initiator, and a water phase containing a stabilizer. Little difference between the raw materials of these two products is noted. There was a porogenic agent in the oil phase in order to make pores in adsorbents when the polymeric adsorbent was synthesized [25]. During the polymerizing period, oil droplets were suspended in the continuous phase (water), due to stirring. After polymerization, spherical products were put in the Soxhlet apparatus and porogenic agents were extracted by organic solvents, resulting in the formation of pores. Microcapsules and Polymer beads were obtained after being dried in a vacuum oven [26].

Accordingly, herein, the main purposes of this work are to: (i) Synthesize a novel polymeric adsorbent which possesses the ability in adsorbing gases and controlling the heat; (ii) optimize the synthesis conditions of the newly synthesized adsorbent; and (iii) test its performance in adsorbing VOCs and controlling the temperature of the adsorption column. We hope this primary work will provide insights into the future of engineering adsorption materials, especially in VOCs removal and recovery by the adsorption-desorption process.

## 2. Materials and Methods 

### 2.1. Materials

Vinylbenzyl chloride (VBC, 30:70 w/w mixture of m-VBC and p-VBC isomers), divinylbenzene (DVB, 80% grade), paraffin wax (melting temperature range of 46–48 °C), and NaCl were purchased from Jiangtian Co. Ltd., Tianjin, China. 1,2-Dichloroethane (DCE), 2,2’-azobisisobutyronitrile (AIBN), toluene, n-heptane, Poly (vinyl alcohol) (PVA), polyvinyl pyrrolidone (PVP), and ferric chloride were purchased from Yuanli Co. Ltd., Tianjin, China. All chemicals were used as received. N2 was purchased from Tianjin Liufang Technology Co. Ltd., Tianjin, China.

### 2.2. Preparation of DVB-Wax Microcapsules

DVB-wax microcapsules were obtained via suspension polymerization in a 250 mL round bottom flask with a mechanical stirrer, reflux condenser, and nitrogen inlet. 140 mL water, 1 g PVP, and 0.68 g PVA were firstly added into the flask as the aqueous phase. The water bath temperature was firstly set at 90 °C for complete dissolution of PVA. Paraffin wax and AIBN were dissolved in DVB and the mixture was transferred into the aqueous phase after complete dissolution of PVP and PVA. The reaction was performed under 450 rpm, stirring at 60 °C for 0.5 h, under nitrogen atmosphere. Then the temperature was gradually increased to 78 °C in another 1.5 h. After the temperature-increasing process, the reaction was kept at 78 °C for 4 h. When small white spheres were clearly observed, temperature was increased to 85 °C to solidify the microcapsules. Microcapsules were then obtained by filtration and washed with water and methanol successively to remove excessive PVP, PVA, and wax. Finally, the microcapsules were dried in a vacuum oven (−0.1 MPa) at 60 °C for 12 h. The reaction was repeatedly conducted with a different ratio of DVB to wax to achieve the highest percentage of encapsulation.

### 2.3. Characterization of DVB-Wax Microcapsules

The surface morphology of microcapsules was examined using a scanning electron microscope (Hitachi SU8020) at 3.0 kV after coating the samples with gold. Differential scanning calorimetric (DSC) analyses were carried out using STA 449 F3 Jupiter from 20 °C to 80 °C at a heating rate of 5 °C/min (3 cycles) to determine the percentage composition of wax in the microcapsule. A solvent resistivity test of microcapsules was done by immersing microcapsules in pure toluene for 1 h. A temperature resistivity test was done by putting the microcapsules in a 120 °C oven for 1 h.

### 2.4. Preparation of VBC-DVB Copolymer Embedded with Microcapsules (VPM)

The synthesis of the Vinylbenzyl chloride-Divinylbenzene (VBC-DVB) copolymer embedded with microcapsules (VPM) was performed in a 150 mL three-neck round bottom flask equipped with a mechanical stirrer, a nitrogen inlet, and a reflux condenser. Water (60 mL), NaCl (0.98 g), PVA (0.36 g), and PVP (0.36 g) were firstly added and mixed as the aqueous phase, using a mechanical stirrer. The water bath temperature was set at 90 °C for complete dissolution of PVA. After 4 h of stirring, the aqueous phase was cooled down to 50 °C. VBC (2.4 mL), DVB (0.6 mL), toluene (2.7 mL), n-heptane (0.3 mL), and AIBN (0.03 g, 1 wt %) were mixed and then transferred into the flask as the organic phase. The mixture was stirred at 180 rpm, 50 °C for 0.5 h under a nitrogen atmosphere. After spherical oil droplets in the mixture were stable as observed, the temperature was gradually raised to 80 °C in another 1.5 h. When the water bath temperature reached 80 °C, DVB-wax microcapsules (1 g) were added into the mixture. Tests have been conducted at the optimized time for the addition of microcapsules and results show that the highest number of embedded microcapsules and good repeatability could be achieved by adding the microcapsules as soon as temperature reached 80 °C. The polymerization process was conducted for another 5 h until the hard-solid beads were synthesized. After the reaction, the products were filtered and washed with hot water and methanol, successively, to remove excessive monomers, NaCl, and stabilizers. The VBC-DVB copolymer beads were finally extracted with ethanol in a Soxhlet apparatus (120 °C) for 12 h, followed by drying for 24 h.

### 2.5. Preparation of Hypercrosslinked VBC-DVB Beads (HVPM)

**Figure 1:**
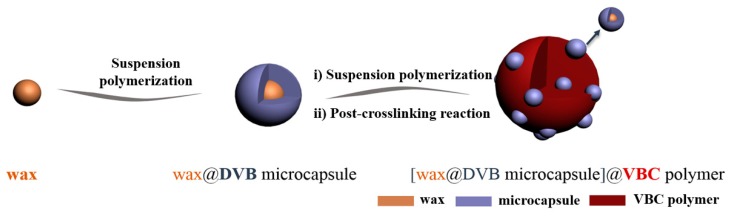
Synthesis procedure for the polymeric adsorbent embedded with divinylbenzene- (DVB)wax microcapsules.

To obtain a microporous hypercrosslinked VBC-DVB polymeric adsorbent embedded with microcapsules (HVPM), a post-crosslinking reaction (Friedel–Crafts reaction) was conducted. VPM, the polymer beads (3 g), were firstly swollen by 1,2-dichloroethane (DCE) (20 mL) overnight to increase their volume for inner-bridging. After the polymer beads were fully swollen, another 80 mL of DCE was added into the mixture and FeCl_3_ (0.6 g), the catalyst, was added. Temperature was then raised to 80 °C. The mixture was stirred at 80 rpm for 8 h to facilitate the inner bridging of polymer beads. After stirring, the hypercrosslinked beads were obtained by filtering the mixture and subsequently washed by water and methanol. The polymer beads were subsequently extracted with ethanol in a Soxhlet apparatus (120 °C) for 12 h, and finally dried in an oven at 60 °C for 6 h until the weight was constant. In conclusion, the overall scheme for the synthesis of HVPM is shown in Figure 1.

### 2.6. Characterization of Polymeric Beads (VPM and HVPM)

The overall profile of VPM and HVPM beads was observed using an Olympus SZX16 microscope. The Fourier transform infrared (FTIR) spectra of VPM and HVPM were obtained using an AVATR360 FTIR spectrometer supplied by Thermo Nicolet (Waltham, MA, USA) under ambient conditions over a wavenumber range of 4000–400 cm^−1^. 

The differential scanning calorimetric (DSC) tests were carried out using STA 449 F3 Jupiter from 20 °C to 80 °C at a heating rate of 5 °C/min, and results were used to determine the heat storage percentage composition of microcapsules in the hypercrosslinked polymeric adsorbent.

The specific surface area and pore size distribution of the adsorbents were calculated by the Brunauer, Emmett, and Teller (BET) and density functional theory (DFT) methods, respectively, via nitrogen adsorption-desorption isotherms, obtained with an ASAP 2460 instrument at −196 °C. The microporous structure was evaluated by a t-plot method. The total pore volume (Vtotal) was estimated to be the liquid nitrogen volume at a relative pressure of 0.99. Before the BET surface area measurement, the adsorbents were degassed at 110 °C for 6 h.

### 2.7. Dynamic Adsorption and Temperature Control Tests

To test the regeneration and reuse performance of synthesized adsorbent HVPM, dynamic adsorption-desorption tests were conducted using toluene as an adsorbate. HVPM was dried in a vacuum oven at 120 °C for 6 h. The regeneration performance of HVPM was investigated using apparatus described in our previous work [27]. Toluene vapor was diluted with nitrogen before it entered the adsorption column (6 mm inner diameter × 3 cm length) at a flow rate of 200 mL/min. By using a mass flow controller, the concentration of toluene vapor in the inlet was fixed at around 13,000 ppm. When the toluene vapor concentration in the outlet was constant and close to the initial concentration, the adsorbent was considered to be saturated by toluene. Desorption was then conducted by a vacuum pump, and a stream of purge gas was introduced into the column as carrying gas. Adsorption-desorption was carried out three times. The masses of the adsorption column loaded with HVPM before and after each cycle were recorded to calculate the adsorption capacities of HVPM.

**Figure 2:**
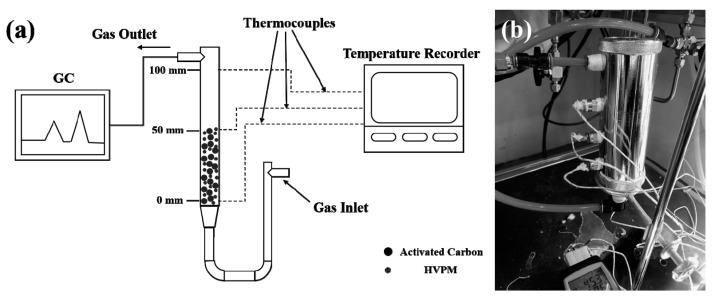
(**a**) Experimental scheme of volatile organic compound (VOC) adsorption with a temperature determination system; (**b**) Image of self-designed adsorption column inserted with thermocouples.

To examine the temperature-control capability of the synthesized adsorbent when it adsorbs high concentration VOCs, dynamic adsorption experiments, designed with an inner-column temperature recording system, were carried out, and toluene was used as an adsorbate. Two adsorption experiments were conducted. The first experiment used activated carbon (40.0 g) as an adsorbent to see the temperature rise during a high concentration toluene-adsorption process. The second experiment used the same amount of activated carbon, (40.0 g) plus HVPM (20.0 g), to verify the temperature-control capability of HVPM. Two adsorbents were evenly mixed then added into the column. The experimental apparatus is shown in Figure 2. Adsorbents were dried overnight in a vacuum oven at 120 °C before measurement. Adsorbent samples were precisely weighed as mentioned above and heated to 45 °C. These samples were then loaded into the adsorption column and filled half the adsorption column (0–5 cm). Because the amount of heat generated was limited during the adsorption for the limited number of adsorbents, the adsorption process was carried out starting at 45 °C. At this temperature, it was helpful for revealing the temperature-control capability of the synthesized polymeric adsorbent because the phase change temperature of encapsulated wax was around 45 °C, shown by the DSC test. Toluene vapor was diluted with nitrogen before it entered the adsorption column (5 cm inner diameter × 10 cm length), containing the adsorbents at 900 mL/min. By using a mass flow controller, the concentration of toluene vapor in the inlet was fixed at around 65,000 ppm. The concentration of toluene in the exit gas stream was monitored with a Flame Ionization Detector. Gas chromatography was used to detect the concentration of VOC. Three thermocouples inserted at different locations in the column (0 cm, 5 cm, 10 cm) were used to detect the real-time temperature of the adsorption column and were connected to the temperature recorder. Tips of the three thermocouples were put in the middle of the column at respective locations. Real-time temperature data was recorded through the temperature recorder. The thermocouple at 0 cm was closest to the inlet of the toluene gas stream. Because the column was not fully filled, the temperature shown by thermocouple at 10 cm was used as a reference.

## 3. Results and Discussion

### 3.1. Characterization of Microcapsules

**Figure 3:**
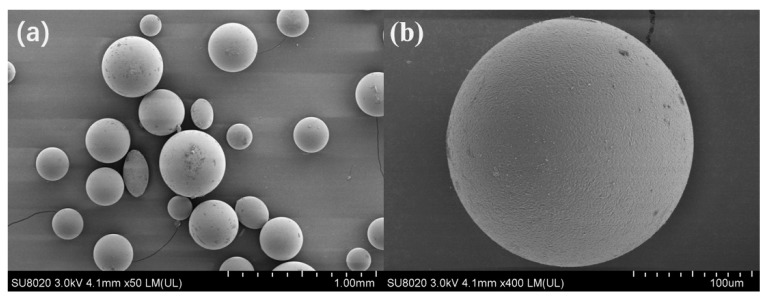
Scanning electron micrographs of (**a**) DVB-wax microcapsules; (**b**) higher resolution image of one randomly chosen microcapsule.

The SEM images of the DVB-wax microcapsules were shown in Figure 3a. The synthesized microcapsule had a good degree of sphericity and the sizes were evenly distributed. The average particle size was determined to be ~150 μm. To further verify the morphology of the DVB-wax microcapsules and surface property, an area was randomly chosen and viewed in higher resolution, as shown in Figure 3b. It is obvious that the microcapsule is a sphere with a smooth surface and no damage or defect. According to the solvent-resistivity and temperature-resistivity tests of DVB-wax microcapsules, no breakage or leakage of wax were observed under lab microscope. It suggests that the microcapsules are solvent-resistive and temperature-resistive. These all provide a solid basis for the further use of DVB-wax microcapsules as synthetic materials in the reaction afterwards.

The DSC tests were conducted for synthesized microcapsules with a different DVB to wax ratio. Microcapsules with the DVB/wax ratio of 2.5:1 could be synthesized with maximum wax encapsulation and no wax leakage. The heat of fusion for pure wax was 147.6 J/g and the counterpart for microcapsules with a DVB/wax ratio of 2.5:1 is 33.8 J/g. The actual value was lower than the theoretical one (42.2 J/g), because some waxes were flushed away together with the washing of PVP and PVA. Another reason was due to the incomplete encapsulation of wax by the microcapsules. It is also found that the phase change temperature of microcapsules was lower than pure wax. It could be attributed to the high latent heat or low heat transfer rate of wax in microcapsules.

### 3.2. Characterization of Polymeric Adsorbents (VPM and HVPM)

**Figure 4:**
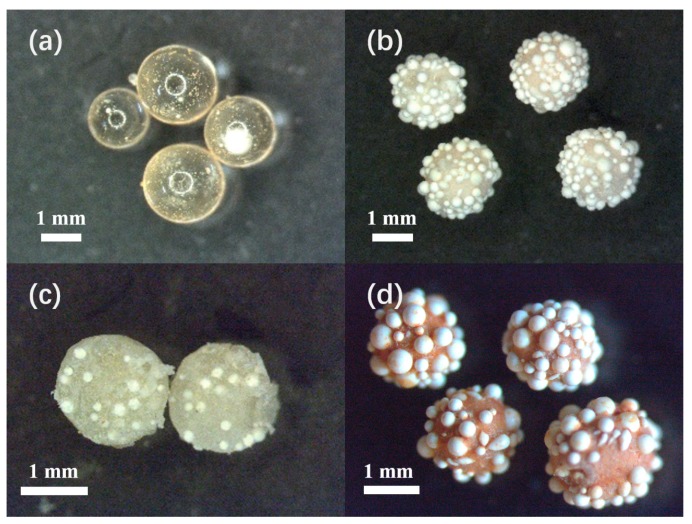
Microscope images of (**a**) Vinylbenzyl chloride-Divinylbenzene (VBC-DVB) copolymer; (**b**) VBC-DVB copolymer embedded with microcapsules (VPM); (**c**) Cross-section profile of one polymer bead; (**d**) Hypercrosslinked VBC-DVB copolymer embedded with microcapsules (HVPM).

The microscope images of VBC-DVB copolymer beads (VPM) and hypercrosslinked beads (HVPM) are presented in Figure 4. Figure 4a is the image of pure VBC-DVB copolymer beads without embedded microcapsules. The beads are transparent, indicating scarcity of micropores. Figure 4b,c are the images of VPM and a cross-section profile of one randomly-chosen bead. As shown in the images, microcapsules are not only dispersed outside the polymeric adsorbents but are also embedded inside. Figure 4d presents the image of HPVM. The color turns brown and opaque because of the mixing of FeCl_3_ into the micropore-dominant structure. For the final products, the average diameter is around 1.5–2.0 mm. The number of microcapsules on each bead is around 40. These characteristics are provided with good heat-storage capability because of a considerable amount of encapsulated wax through the microcapsules.

**Figure 5:**
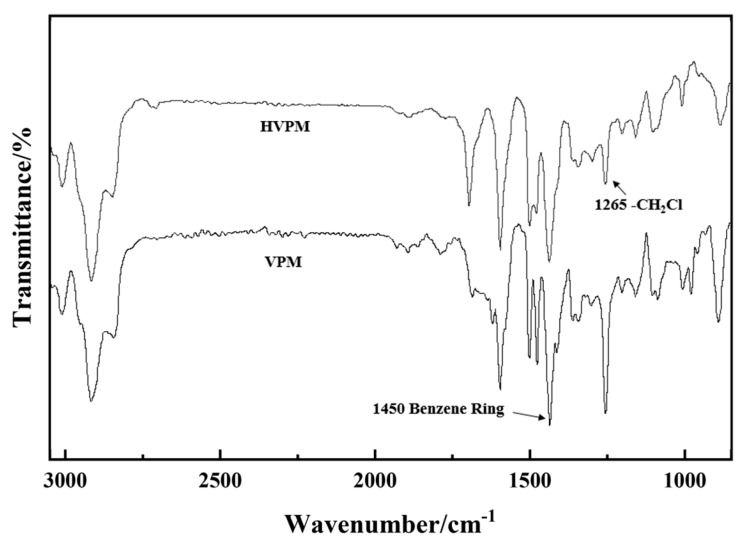
FTIR spectra for polymeric adsorbent before and after post-crosslinking reaction.

Figure 5 presents the FTIR of VPM and HVPM. The sharp peak at 1265 cm^−1^ indicates the presence of a CH_2_-Cl group from VBC. In the IR spectra obtained for HVPM, the characteristic peak almost disappeared, due to the inner bridging of the polymer beads. CH_2_-Cl groups are connected, resulting in large amounts of micro/mesopores. The spectra prove the completion of post-crosslinking reaction. The N_2_ adsorption-desorption isotherms of polymeric adsorbents without post-crosslinking reaction (VPM) also proves the above conclusion. The isotherm is not shown because it has almost no sign of gas adsorption and the reason is that VPM is mainly composed of mesopores and macropores before the post-crosslinking reaction.

**Figure 6:**
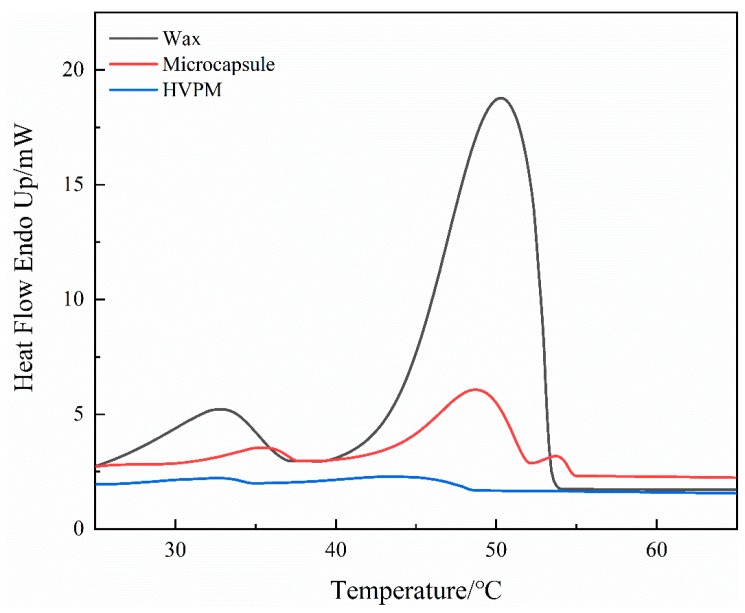
Comparison of the differential scanning calorimetric (DSC) thermograms for wax, DVB-wax microcapsules, and HVPM.

The DSC results for pure wax, microcapsules, and polymer beads are shown in Figure 6 and Table 1. The fusion heat of microcapsules is 33.8 J/g and the counterpart for polymer beads is 11.1 J/g. It suggests that the percentage of microcapsules in polymer beads is around 32.8%, which corresponds to the high percentage of embedment viewed from lab microscope images. The fusion heat of HVPM indicates the embedment of microcapsules, which results in a thermal energy storage capability. The practical effect in controlling inner-column temperature is to be shown in the next section.

**Figure 7:**
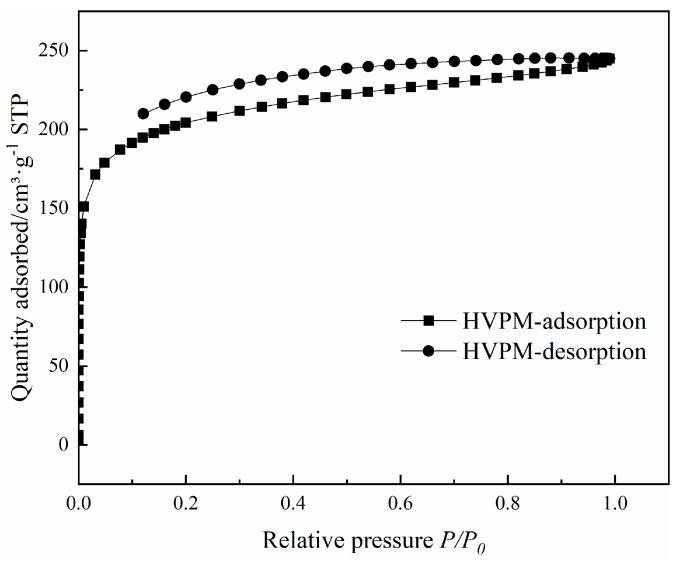
Nitrogen adsorption-desorption isotherms of HVPM.

The N_2_ adsorption-desorption isotherms of HVPM at 77 K are demonstrated in Figure 7. It is observed that at lower relative pressure (P/P_0_ < 0.05), the amount of adsorbed nitrogen increases sharply as the relative pressure increases. At high relative pressure, however, no further significant increase was found. According to the International Union of Pure and Applied Chemistry (IUPAC) classification, this kind of isotherm can be classified as type I, proving the existence of micropore-dominant structure. Relative physical properties are listed in Table 2.

**Figure 8:**
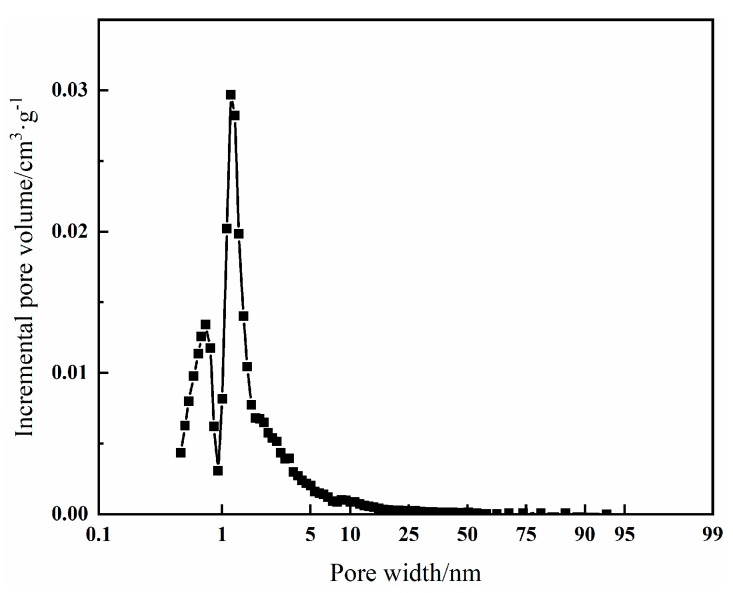
Pore size distribution of HVPM.

As shown in Table 2, it is clear that the hypercrosslinked adsorbent has a relatively large specific surface area (665 m^2^/g) and a micropore-dominant structure (micropore volume 0.24 cm^3^/g, total pore volume 0.38 cm^3^/g), which was beneficial for the adsorption process. The pore size distribution of the hypercrosslinked polymeric adsorbent, calculated by applying the density functional theory (DFT) to the N_2_ adsorption-desorption isotherms at 77 K, is shown in Figure 8. It was found that the adsorbent possesses both micro-pores (0–2 nm) and meso-pores (2–50 nm). The pores were dominant in micro-pores, which were formed through the post-crosslinking reaction. These micropores were mainly produced by the methylene bridges in the pores. The meso-pores were mainly generated by the diluents and uncompleted post-crosslinking reaction. The formation of small meso-pores is attributed to the use of a high proportion of toluene to heptane (9/1, vol/vol) as diluent [28]. The presence of both micro-pores and meso-pores would ensure good adsorption capability and high desorption efficiency, which guarantee ideal adsorption outcome and renewable use.

### 3.3. Dynamic Adsorption and Temperature Control Tests

**Figure 9:**
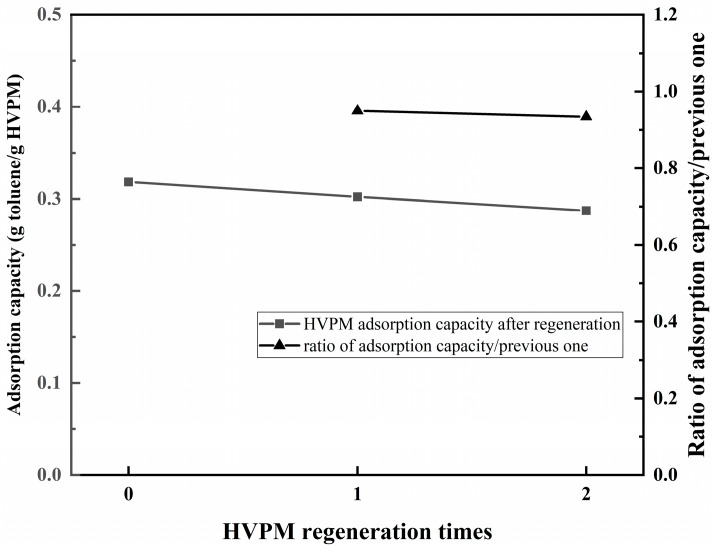
Regeneration and reuse performance of HVPM.

Dynamic adsorption-desorption tests for HVPM were firstly conducted using a small-scale apparatus to test the regeneration and reuse performance of HVPM. Results were shown in Figure 9. For the first-time adsorption of toluene, HVPM had an adsorption capacity of 0.32 g toluene/g HVPM. Three adsorption-desorption cycles were subsequently conducted, and the synthesized adsorbent only showed a 0.03 g/g decrease in adsorption capacity. The ratio of the adsorption capacity and the previous adsorption capacity was over 0.93, which also suggests good regeneration performance.

**Figure 10:**
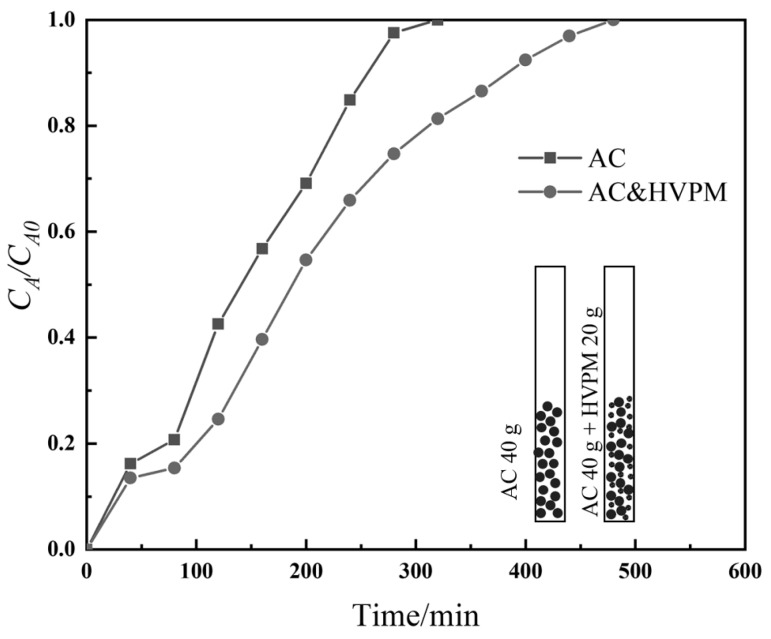
Breakthrough adsorption curves of (i) activated carbon (AC); (ii) AC and HVPM toward toluene. (Temperature: 45 °C; feed flow rate: 0.90 L/min; initial toluene concentration: 65,000 ppm).

Using a self-designed adsorption column, which enables real-time inner-column temperature recording, temperature-control capability of the synthesized HPVM was tested. Two adsorption tests were carried out using (i) activated carbon (40 g), (ii) activated carbon (40 g) and HVPM (20 g), as adsorbents. Toluene was used as adsorbate. The temperature of the adsorbents and inlet gas was set at 45 °C, using an oven and water bath, respectively. The breakthrough curves of the two experiments are shown in Figure 10. Initial toluene concentration and flow rate were both high so there was toluene found at the gas exit at the beginning. The adsorption capacities of activated carbon and HVPM were 0.31 and 0.21 g/g, respectively. The value was determined by weighing the adsorbents after the adsorption process. The result shows the considerable adsorption capability of HVPM.

**Figure 11:**
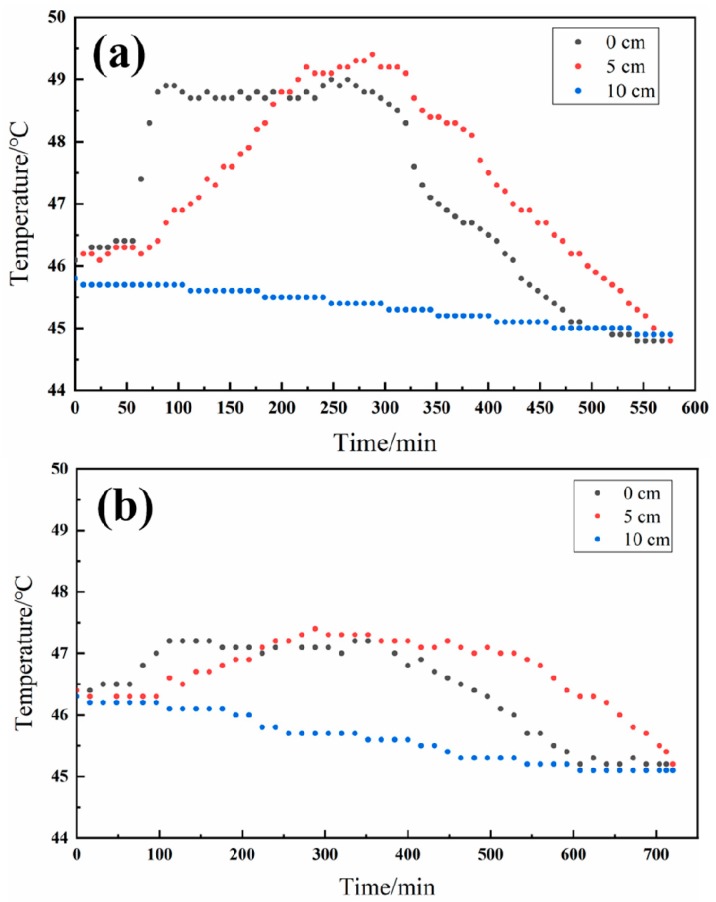
(**a**) Inner-column temperature change during toluene-adsorption process using activated carbon as adsorbent; (**b**) inner-column temperature change during toluene-adsorption process using activated carbon and HVPM as adsorbent.

Figure 11a shows the real-time inner-column temperature, using activated carbon as an adsorbent. Adsorption started at 0 min and, at 0 cm, the temperature started to increase because of released heat due to the adsorption. As the adsorption occurred from bottom to top of the column, the temperature at 5 cm subsequently increased because of heat transferred from the lower position and the released heat of the adsorption. The highest temperature in the column reached near 49.5 °C. As the activated carbon in the lower position reached saturation adsorption, the temperature at 0 cm slowly decreased. Finally, as adsorption was completed, the temperature at different locations decreased. The onset time of the temperature-decreasing trend at 5 cm corresponded to the saturation adsorption time (around 320 min) indicated by the breakthrough curve in Figure 10. The final temperature was lower than the initial temperature because of the heat transfer between column and air. 

Figure 11b shows the real-time inner-column temperature using activated carbon and HPVM as an adsorbent. A similar thermal behavior was observed, compared to Figure 10a. However, the final highest temperature was around 47.4 °C. Around 1.2 °C, the temperature rise occurred in the second experiment, in contrast with 3.2 °C in the pure activated carbon adsorption experiment, indicating a 62.5% decrease in temperature rise. The large difference in temperature rise is because the wax in HVPM stores the heat released from adsorption as it undergoes phase transition. The temperature-buffer characteristic of HVPM also results in a lower gradient, both in temperature rise and temperature drop, as seen in Figure 11b. Even for this small-scale adsorption test, the temperature rise was high in the column of activated carbon adsorption, as shown in Figure 10a, and the synthesized HVPM shows a capability of controlling the inner-column temperature during the adsorption process, as expected.

## 4. Conclusions

The idea of embedding PCM microcapsules in adsorbent was innovatively proposed in this study in order to combine the adsorption capability of polymeric adsorbent with the heat storage capability of PCM microcapsules. We found that for the maximum embedment of DVB-wax microcapsules, the optimal time for the addition of microcapsules was when the reaction temperature reached 80 °C. The dynamic adsorption and temperature control experiments proved the capabilities in VOC adsorption and temperature control. This new polymeric adsorbent material could provide new insights on solving the problem of fire and explosion risks during high concentration VOCs adsorption processes.

## Figures and Tables

**Table 1:** DSC data for wax, DVB-wax microcapsules, and HVPM.

	T_onset_^a^/°C	T_end_^b^/°C	T_onset_^c^/°C	T_end_^d^/°C	△H ^e^/J·g^−1^	Percentage of Precursor ^f^/%
Wax	27.0	37.3	39.1	54.4	147.6	
Microcapsule	31.3	38.7	38.9	51.8	33.8	22.9
HVPM	32.0	34.6	35.0	48.2	11.1	32.8

a: Wax solid-solid phase change onset temperature; b: Wax solid-solid phase change end temperature; c: Wax solid-liquid phase change onset temperature; d: Wax solid-liquid phase change end temperature; e: Heat of fusion of wax/microcapsule/HVPM; f: Percentage of precursor (wax in microcapsule, microcapsule in HVPM).

**Table 2:** Selected maximum BET surface areas and the related parameters of HVPM.

Sample	S_BET_^a^/m^2^·g^−1^	S_micro_^b^/m^2^·g^−1^	S_meso_^c^/m^2^·g^−1^	V_micro_^d^/cm^3^·g^−1^	V_meso_^e^/cm^3^·g^−1^	V_total_^f^/cm^3^·g^−1^
Value	665	501	162	0.24	0.08	0.38

Note: a: S_BET_: Specific surface area; b: S_micro_: Specific microporous surface area; c: S_meso_: Specific mesoporous surface area; d: V_micro_: Micropore volume; e: V_meso_: Mesopore volume; f: V_total_: Total pore volume.
